# Sinskey Hook-Guided Mechanical Peeling of Primary Posterior Capsular Plaque During Cataract Surgery

**DOI:** 10.7759/cureus.107205

**Published:** 2026-04-16

**Authors:** Md Iftekher Iqbal

**Affiliations:** 1 Department of Ophthalmology, Bangladesh Eye Hospital, Dhaka, BGD; 2 Department of Glaucoma, Ispahani Islamia Eye Institute and Hospital, Dhaka, BGD

**Keywords:** nd:yag laser capsulotomy, posterior capsular opacity, posterior capsulotomy, posterior subcapsular cataract, sinskey hook

## Abstract

Primary posterior capsular plaque may be encountered during cataract surgery and can compromise the visual axis. Although Nd:YAG laser capsulotomy effectively treats postoperative posterior capsular opacification, it carries recognized risks, and intraoperative management may be preferable in selected cases. This report describes a simplified Sinskey hook-guided mechanical peeling technique performed before intraocular lens implantation. In a single adult case, plaque separation was achieved through controlled tangential glide-dissection beneath the plaque using a Sinskey hook, followed by forceps extraction. An ophthalmic viscosurgical device was used only for anterior chamber maintenance. The posterior capsule remained intact, allowing uncomplicated in-the-bag intraocular lens implantation. At three months, the visual axis remained clear without inflammation, intraocular pressure elevation, or need for Nd:YAG capsulotomy. This technique provides a simple, instrument-efficient intraoperative option for selected posterior capsular plaques.

## Introduction

Primary posterior capsular plaque is a dense fibrotic opacity adherent to the posterior capsule that may be identified intraoperatively during cataract surgery. Unlike postoperative posterior capsular opacification (PCO), these plaques are present at the time of surgery and may directly obstruct the visual axis if left untreated [1,2].

Nd:YAG laser capsulotomy remains the standard treatment for visually significant PCO; however, it is associated with well-recognized complications, including transient or sustained intraocular pressure elevation, cystoid macular edema, retinal detachment, and intraocular lens (IOL) pitting [3-6]. In selected cases, intraoperative management of posterior capsular plaque may therefore be advantageous to achieve immediate visual axis clarity and defer or avoid early laser intervention [7-9].

Several surgical approaches have been described for intraoperative plaque management, including irrigation-aspiration polishing, viscodissection-assisted removal using a Sinskey hook, retinal forceps peeling, and primary posterior continuous curvilinear capsulorhexis when plaque removal is not feasible [7,9-11]. Each technique has inherent limitations related to capsule safety, surgical complexity, or instrument availability.

The present report describes a simplified mechanical peeling technique using a Sinskey hook to develop a separation plane beneath the plaque, followed by forceps extraction, performed before IOL implantation.

## Technical report

Ethical review and approval were waived because this report is based on routine clinical care and does not constitute human subject research. Written informed consent was obtained from the patient for publication of clinical details and images. The study adhered to the tenets of the Declaration of Helsinki.

Case presentation

**Figure 1:**
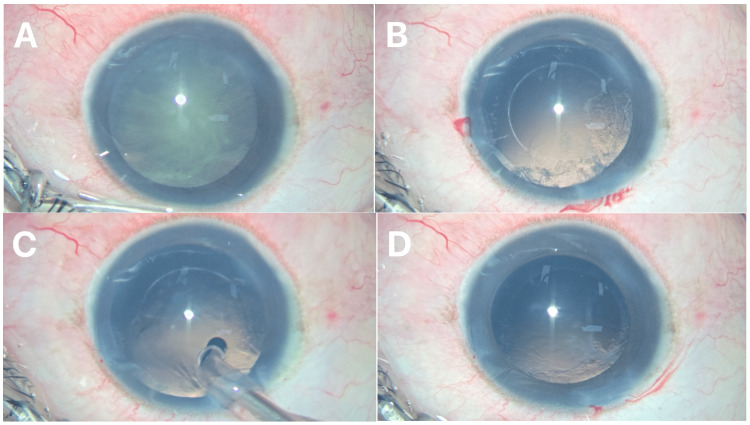
Peroperative images. (A) Dense posterior subcapsular cataract and (B) thick posterior capsular plaque (C, D), which was not removed after polishing with an irrigation-aspiration probe.

A single adult patient with posterior subcapsular cataract undergoing routine phacoemulsification surgery was noted intraoperatively to have a dense, centrally located posterior capsular plaque following complete cortical cleanup (Figure 1, Panels A, B). The plaque involved the visual axis and was considered unsuitable for simple irrigation-aspiration polishing (Figure 1, Panels C, D).

Description of technique

After standard phacoemulsification and complete cortical cleanup, a dense posterior capsular plaque was identified.

Step 1: Chamber Stabilization

**Figure 2:**
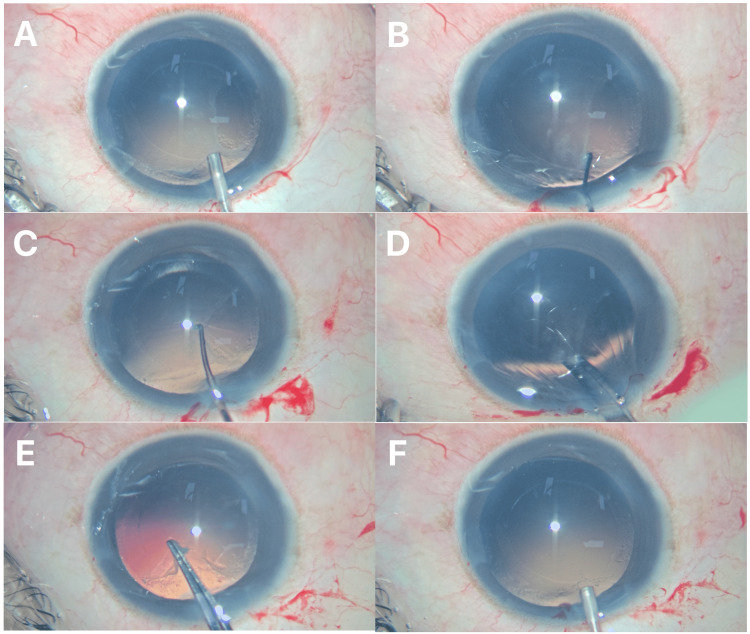
Sinskey hook-assisted posterior capsular plaque peeling. (A) Anterior chamber stabilization with an ocular viscosurgical device. (B) Posterior capsular plaque edge identification and lifting with a Sinskey hook. (C) Mechanical glide-dissection using a Sinskey hook. (D) Cutting fibrous plaque edge with Vannas’s scissors. (E) Grasping and removal of posterior capsular plaque using capsulorhexis forceps. (F) Intact posterior capsule visible.

A cohesive ophthalmic viscosurgical device (OVD) was injected to maintain a stable anterior chamber without excessive posterior capsule distension (Figure 2, Panel A).

Step 2: Edge Identification and Lifting Maneuver

Using the tip of a Sinskey hook, a peripheral edge of the plaque was gently elevated with minimal vertical force (Figure 2, Panel B). Vertical traction was minimized to avoid stress on the posterior capsule.

Step 3: Mechanical Glide-Dissection

Once an edge was engaged, the Sinskey hook was advanced tangentially beneath the plaque, maintaining close apposition to the posterior capsule. Short, controlled side-to-side sweeping movements were used to propagate a mechanical separation plane between the plaque and posterior capsule (Figure 2, Panel C). OVD was used only for chamber maintenance and not injected beneath the plaque.

Step 4: Plaque Extraction

As the plaque was thick enough to peel, Vannas’s scissors were used to carefully cut it into a thicker point (Figure 2, Panel D). After adequate mobilization of the plaque, the free edge was grasped with capsulorhexis forceps and removed in a controlled manner (Figure 2, Panel E).

Step 5: Capsule Assessment

Posterior capsule integrity was confirmed under direct visualization (Figure 2, Panel F).

Key safety principles included maintaining tangential traction, avoiding central pulling forces, and abandoning the maneuver if a safe dissection plane could not be established. Video 1 shows the detailed surgical procedure of this technique.

**Video 1:** Sinskey hook-assisted posterior capsular plaque peeling.

**Figure 3:**
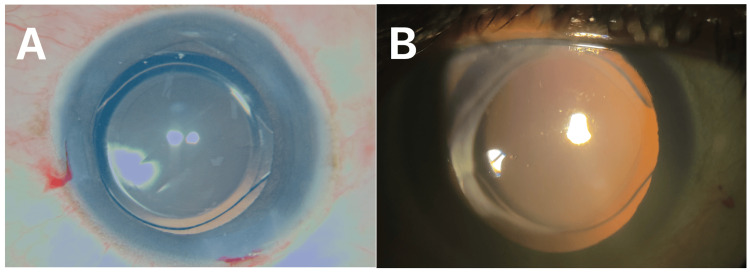
Intraoperative and postoperative images. (A) Uncomplicated in-the-bag intraocular lens implantation during surgery. (B) Stable, well-centered intraocular lens and no posterior capsular opacity during three-month postoperative follow-up.

Complete removal of the posterior capsular plaque from the visual axis was achieved without posterior capsular rupture, vitreous prolapse, or zonular compromise. The posterior capsule remained intact, allowing uncomplicated in-the-bag IOL implantation (Figure 3, Panel A).

At the three-month postoperative follow-up, the visual axis remained clear (Figure 3, Panel B). There was no clinically significant anterior chamber inflammation, intraocular pressure elevation, or requirement for Nd:YAG capsulotomy.

## Discussion

The risk of posterior capsule rupture continues to pose a challenge in the management of primary posterior capsular plaque during cataract surgery [2]. The viscodissection-assisted techniques rely on the hydraulic separation of the plaque, whereas retinal forceps peeling techniques necessitate posterior segment instrumentation and expertise [7,12]. When plaque removal is risky or ineffective, primary posterior capsulotomy may be performed; however, it intentionally compromises posterior capsule integrity [7,9,11].

The technique outlined here is distinct in that plaque separation is predominantly accomplished via controlled mechanical glide-dissection with a Sinskey hook, with OVD employed solely for maintaining chamber stability. This approach emphasizes tangential forces, avoids hydraulic plaque separation, and relies exclusively on standard anterior segment instruments. Undertaking the maneuver before IOL implantation facilitates clear visibility and diminishes the likelihood of IOL-associated problems [9,11].

It is important to distinguish intraoperative plaque removal from long-term PCO prevention [12]. Available evidence suggests that the presence of posterior capsular plaque does not necessarily increase long-term PCO rates following cataract surgery [2,8,12-16]. The primary goal of this technique is to clarify the visual axis immediately and potentially avoid or defer early Nd:YAG capsulotomy in selected cases.

This report describes a single case with a short-term follow-up. The technique should be reserved for carefully selected cases where a safe separation plane can be established. Dense plaques firmly integrated with the posterior capsule may still require alternative management strategies, including delayed Nd:YAG capsulotomy or primary posterior capsulotomy.

## Conclusions

Sinskey hook-guided mechanical peeling provides a simple and controlled option for intraoperative removal of selected posterior capsular plaques during cataract surgery. When performed with careful attention to tangential dissection and chamber stability, the technique allows preservation of posterior capsule integrity and uncomplicated in-the-bag IOL implantation. Further experience is required to define its broader applicability.
